# Modelling water table depth variation in Southeast Asia forested peatlands using satellite rainfall data

**DOI:** 10.1038/s41598-026-64641-2

**Published:** 2026-08-24

**Authors:** A. Hooijer, R. Vernimmen

**Affiliations:** Data for Sustainability, Axel, 4571 AK The Netherlands

**Keywords:** Regional rainfall patterns, Tropical peatland water table depth, Water table depth model, SE Asia, Indonesia, Malaysia, Climate sciences, Ecology, Ecology, Environmental sciences, Hydrology

## Abstract

Carbon emissions from drained tropical peatlands are of global concern. Restoration relies on ‘rewetting’ to reduce water table depth (WTD), aiming to recreate natural ecosystem conditions. However, in Southeast Asia little is known about the actual WTD in undisturbed peatlands, to inform restoration targets. To fill this knowledge gap, we have modelled WTD in forested peatlands, yielding a 25-year record of indicative daily WTD, using IMERG satellite rainfall data. The model was calibrated with WTD records of undrained peat swamp forest (PSF) sites in four different regions. The resulting maps reveal a large geographic variation in natural peatland hydrology, with lowest 3-month minimum rainfall ranging from above 250 mm along northern coastlines to below 40 mm in southern parts. This causes a wide range in peatland WTD regimes, with mean annual minimum WTD varying from − 0.3 m to − 0.9 m. In wettest regions the WTD is never below − 0.5 m, which occurs frequently in other areas. We propose that these regional, seasonal and interannual variations patterns in rainfall and WTD should be reflected in peatland land- and water management targets and in interpretation of studies of hydrology, carbon cycle and fire risk in peatlands.

## Introduction

Peatlands in SE Asia have been deforested and drained at a high rate in recent decades, with only around 29% of the 15.7 Mha in Sumatra, Borneo and Peninsular Malaysia remaining somewhat forested by 2015 and only 6.4% considered ‘pristine’^1^. Nearly all deforested areas are drained, for removal of timber or for agricultural water management, and often for a combination of the two. Many areas that remain forested also have their hydrology altered to some degree, often by ditches and tracks for transport of harvested logs^2^, or by nearby plantation areas that can impact water levels over distances beyond 1 km^3^.

The position of the water table below the peat surface is a measure of moisture content of the topsoil, which determines the ability of air to enter the soil. This condition is considered the main control on biological oxidation of the peat and on associated peat loss that results in both carbon emission to the atmosphere, that is of global climate concern^4^, and subsidence of the peat surface that causes flooding and agricultural production loss^5–7^. Peatland water table depth (WTD) therefore is a key parameter in determining carbon emission^8,9^ and also controls the availability of dry peat to fuel fires^10,11^. The major peatland fires that occurred in recent decades, producing haze that is a regional environmental concern, took place in periods and areas with low water tables.

Initiatives to reduce peatland carbon emissions in SE Asia, as elsewhere, therefore always include a rewetting component that involves blocking of canals to raise water levels and reduce or even reverse the impacts of drainage. However, no clear understanding exists of what the ‘natural’ or ‘original’ WTD would be that can inform an optimum rewetting target. Insofar such targets are currently considered, they are uniform and do not take into account geographic or temporal variations. For example, in an effort to reduce fire risk and carbon emission, Indonesian Government has introduced regulations to avoid peatland water tables falling below − 0.4 m^12^. However, while all peatlands in the region are created by ‘peat swamp forest’ (PSF) which is a type of tropical rainforest, rainfall is known to be highly variable geographically^13^ and WTD could be equally variable as a consequence. Applying a uniform WTD target may not be the best approach to restoring near-natural conditions in all regions.

To assess whether a variable WTD target in PSF conservation and restoration is required, we have created 25-year synthetic records of forested peatland WTD by applying satellite rainfall data to a model. Such data are collected globally since 1998 and were found to be more accurate than most ground measurements in SE Asia especially in the identification of drought conditions^13^; moreover they offer full coverage of any location in contrast to sparsely distributed ground measurements. The recent publication of several long-term field records of WTD in nearly undrained forested peatlands across the region^3,14–16^ provides high-quality WTD data for different climate zones that allows confident model calibration.

The model was applied to Peninsular Malaysia and the islands of Sumatra and Borneo that contain most peatland in SE Asia. Daily WTD results were summarized into key statistics that characterize peatland hydrological regime, and presented in maps and tables. While the primary aim is to support definition of rewetting targets for forested and degraded peatland areas where restoration is planned, we expect these results to also be useful in minimizing fire risk and carbon emission in drained peatlands where no full rewetting is planned. Moreover, the results will improve understanding of the representativeness of published WTD and carbon emission records. Finally, quantifying variations in rainfall and hydrological regime will help understanding variations in ecosystem functioning and associated past peat accumulation rates.

## Methods and data

### Peatland extent

**Fig. 1:**
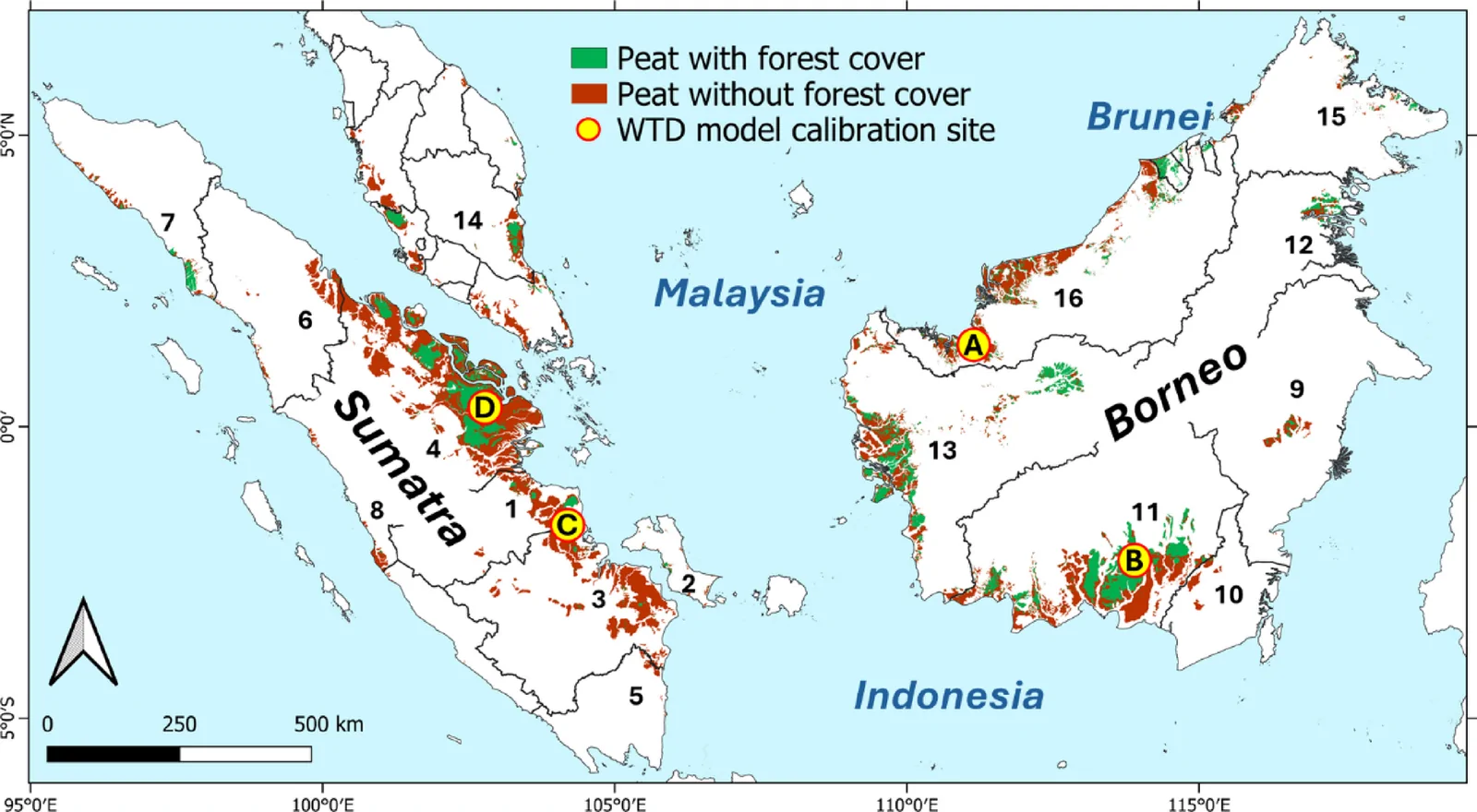
Overview map of study area: peat extent, 2024 forest extent^21^ and calibration site locations (Table 1). Numbers refer to geographic units (Table 2).

The model area covers the peatlands of Peninsular Malaysia, Sumatra and Borneo (Fig. 1). For the peatland extent for Sumatra and Kalimantan (Indonesian part of Borneo Island) we used the map published by the Indonesian Ministry of Agriculture^17^. For Malaysia we used maps provided by the European Digital Archive of Soil Maps^18^ following the approach described by Miettinen and Liew^19^. For Brunei we used the Anderson and Marsden forest type map^20^.

### Satellite rainfall data

Satellite rainfall maps at 0.1-degree horizontal spatial resolution (~ 10*10 km) were created from version 07B of the IMERG daily research dataset^22^. The full daily record available covers the period of 1998 to 2024. For the forested peatland WTD model calibration sites, the time series for the matching satellite rainfall grid cell was applied. For analysis of rainfall regime, rainfall data were aggregated over peat areas at the level of Indonesian Provinces, Malaysian States and the country of Brunei.

### Calibration records of tropical forested peatland WTD

For model calibration, four long-term records of field measurements from well-described research sites on intact or nearly-intact forested peat of over 5 m thickness were applied. The records cover at least 5 years and study sites are well distributed geographically and over climate zones, from Sarawak in North Borneo^14^ (8 years) to Central Kalimantan in south Borneo^16^ (16 years) and from Riau in the northern part of Sumatra^15^ (6 years) to South Sumatra^3^ (5 years). Three of these published records present monthly data^3,14,15^ while one record consisted of daily data from which we selected monthly values^16^. Except one record for which we read values from a graph^14^, data were obtained in digital format with exact date indication. Apart from the Central Kalimantan data^16^ that were derived from a single measurement location (‘dipwell’), WTD values are averaged over same-day measurements from 2 or more locations that are at least 500 m apart and over 1.5 km away from the nearest known drainage canal. The records used from the Riau^15^ and Central Kalimantan^16^ sites are exactly as in publications, whereas for the Sarawak^14^ site we used the mean values calculated over 2 records for different PSF types on deep peat, excluding a record near the river side. For the South Sumatra^3^ site we used the last 4.5 years of the published record plus an additional 6 months of new data up to late 2023, to include only conditions after site rewetting of the wider restoration area was completed.

For comparison, we identified a further 8 records of forested peatland WTD measurements in PSF, generally within 100 km from the calibration sites. These records were mostly in older publications with less detailed description of location and conditions. They were also shorter in duration (one to three years), and on sites where drainage impact cannot be excluded. These records were therefore used only to visually demonstrate the representativeness of calibration records, and to discuss spatial variability in WTD within regions.

### Tropical forested peatland WTD model

We have applied a model that presents a simplified concept of hydrology, ensuring regional applicability by minimizing the need to quantify parameters based on the limited knowledge base available for most PSF types in the region. In this concept, water table fluctuation in the peat is controlled by rainfall, and losses to evapotranspiration and water outflow, moderated by the peat ‘specific yield’ that is related to porosity and determines how a change in water storage translates to a change in WTD.

A model was applied and implemented in Python code to construct records of peatland water table depth since 2000. The model was initiated from 1998 onwards, applying an initial WTD value of ‘0’ to ensure a valid water table depth by the start of 2000. Daily satellite rainfall data are input to a ‘bucket’ water balance model that is optimized to yield the best fit with field measurements:

1 $$WTD_{t} = WTD_{{t - 1}} + 1/Sy*\left( {P{-}\left( {ET + WF} \right)} \right)/1000$$

And:

2 $$if\,WTD_{{t - 1}}> 0,{\text{ }}then\,WTD_{t} = 0$$

Where:

*WTD*_*t−1*_ = the water table depth on the previous day [m]

*Sy* = the ‘specific yield’ or ‘storage coefficient’, a peat characteristic linked to porosity, that determines the change in WTD resulting from a change in soil water storage [between 0 and 1].

*P* = rainfall [mm d^− 1^]

*ET + WF* = evapotranspiration (*ET*) plus lateral flow of water to streams or drainage canals (WF). Different *ET + WF* values are applied to WTD ranges below and above ‘0’ [mm d^− 1^].

### Tropical forested peatland WTD model parameter values

Apart from rainfall, which is a dynamic parameter that we derive from satellite records, the other model parameters are kept constant in time and across the region, for all four calibration sites. We estimate these values from published studies where available.

### Evapotranspiration

*ET* for SE Asia tropical PSF is relatively well studied, with a range of 4.3 to 5.2 mm d^− 1^ (1570–1899 mm yr^− 1^) being reported^23-25^. As *ET* is largely controlled by the vegetation transpiration component, which is a function of plant growth, it is a relatively confined parameter within a limited range. In these studies, *ET* implicitly or explicitly includes the rainfall interception component.

### Lateral water flow

The rate of lateral surface- and groundwater flow (*WF*) in intact PSF is controlled by water table gradients that are typically below 1 m km^− 1^ in intact peat swamps^26,27^. Because of this very low gradient, *WF* is sometimes considered ‘negligible’ when the water table is more than 0.1 m below the peat surface^26^. An assessment by Cobb et al.^27^ also concludes that groundwater flow rate, as controlled by transmissivity of the peat, is very low as long as the water table is below the peat surface, but increases rapidly above it; from their numbers we estimate a mean value around 0.5 mm d^− 1^ below the peat surface. In their conceptual model, flow over the peat surface is considered a form of groundwater flow as the water is confined to isolated pools, being able to move laterally only through the peat in hummocks and ridges separating the pools, with flow rates rapidly increasing as the water table rises further above the peat surface.

### Combined ET + WF

In our simplified model, we apply a constant water loss of 5 mm d^− 1^ when the water table is below the peat surface, based on combined literature-based values of ~ 4.5 mm d^− 1^ for *ET* (including interception) and an estimate of ~ 0.5 mm d^− 1^ for *WF* below the peat surface.

The remaining *WF* component when the water table is above the peat surface must be considerably higher but is extremely variable in time, as it occurs mostly during and shortly after heavy rainfall. It is therefore estimated through model optimization, as a ‘loss factor’ to fit calibration records.

### Specific yield

*Sy* of the porous peat matrix is highly variable with the position within the peat profile. Hooijer^26^ reports a value of 0.29 when WTD is below − 0.1 m but 0.71 above this level, while Cobb et al.^27^ indicate values dropping below 0.15 when the WTD is below − 0.2 m but rapidly increasing to above 0.3 towards the peat surface. Assessment of data presented by Deshkmukh et al.^15^ suggests a value around 0.56 across the entire water table depth range. Based on these scarce published values we determine a fitting *Sy* value below the peat surface through model optimization, within the published range of 0.3 to 0.6.

### Further note on parameter optimization

It is worth noting that the effect on modelled WTD of lowering *Sy* is the same as increasing *ET + WF*, and vice versa. We have therefore not attempted to determine absolute values for these two hydrological parameters but have instead optimized a combination that is plausible in view of published values, while allowing us to simulate field measurements as accurately as possible.

### Tropical forested peatland WTD model calibration approach

The WTD model was calibrated by minimizing to zero the mean of the mean differences between measured and modelled same-date monthly WTD values for the four calibration sites. In parallel, the mean of the coefficients of determination (R^2^) between modelled and measured WTDs across the four sites was maximized, while also aiming to have regression lines approach 1:1 as closely as possible. Calibration was done manually, by working through a matrix of *ET + WF* (when the water table is above the peat surface) and *Sy* values, identifying the combination that yielded the best model fit. Above-surface *ET* + *WF* values were increased from 10 to 100 mm d^− 1^ in steps of 5 mm d^− 1^, and *Sy* values ranged from 0.3 to 0.6 in steps of 0.01; all plausible combinations of these two parameters were tested.

As the aim was to create a WTD simulation model for conditions when WTD is below the peat surface, and considering that WTD above the peat surface represents inundation that is a function of rapid lateral inflows and outflows through the complex hummock-hollow microtopography typical of the PSF floor, WTD values above the peat surface were set at ‘zero’ when determining the difference between measured and modelled WTD. When determining the R^2^, WTD values above − 0.05 m were excluded.

### Definition of hydrological regime

#### Rainfall regime

Variations in rainfall, calculated from daily data, were summarized into annual parameters most relevant to the nature of the hydrological regime in peatland, over the 25-year record. We calculated annual mean, minimum and maximum amounts, as well as annual 3-month minimum values over a moving 91-day window. A further drought indicator involved calculation of the number of consecutive days over which the antecedent 30-day rainfall was below 100 mm, as proposed by Kume et al.^28^.

#### WTD regime

Variations in modelled WTD were calculated and mapped on the ~ 10*10 km rainfall grid, over the 25-year record, for all peatland including non-forested (but potentially to be rewetted) areas. Parameters included mean annual WTD, mean annual minimum WTD, minimum WTD across the record, mean period of time per year with WTD below − 0.5 m, longest period of time per year on record with WTD below − 0.5 m, mean period of time per year with WTD below − 1 m, and longest period of time per year with WTD below − 1 m. For analysis of hydrological regime, WTD values determined at ~ 10*10 km horizontal spatial resolution were aggregated over peat areas at the level of Indonesian Provinces, Malaysian States and the country of Brunei.

## Results

### Geographic and temporal variation in rainfall

Mean annual mean rainfall over calibration areas varied from 2441 to 3727 mm yr^− 1^ during calibration periods, and from 2490 to 3726 mm yr^− 1^ across the 25-year record (Table 1). The small difference between values for the two record lengths suggest that the duration of calibration periods, of at least 5 years, is sufficient to be representative of long-term conditions.

**Table 1:**
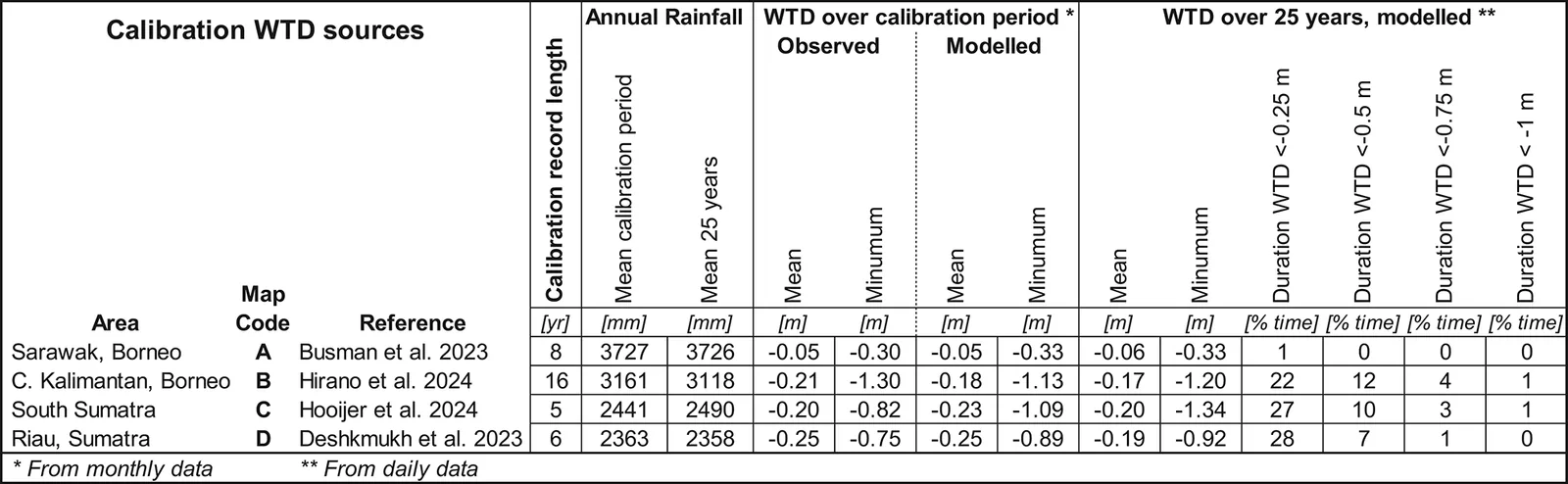
Summary statistics of annual rainfall and observed and modelled WTD for calibration locations, over calibration periods and over 25-year record (2000–2024). Map code locations are shown in Fig. 1.

**Fig. 2:**
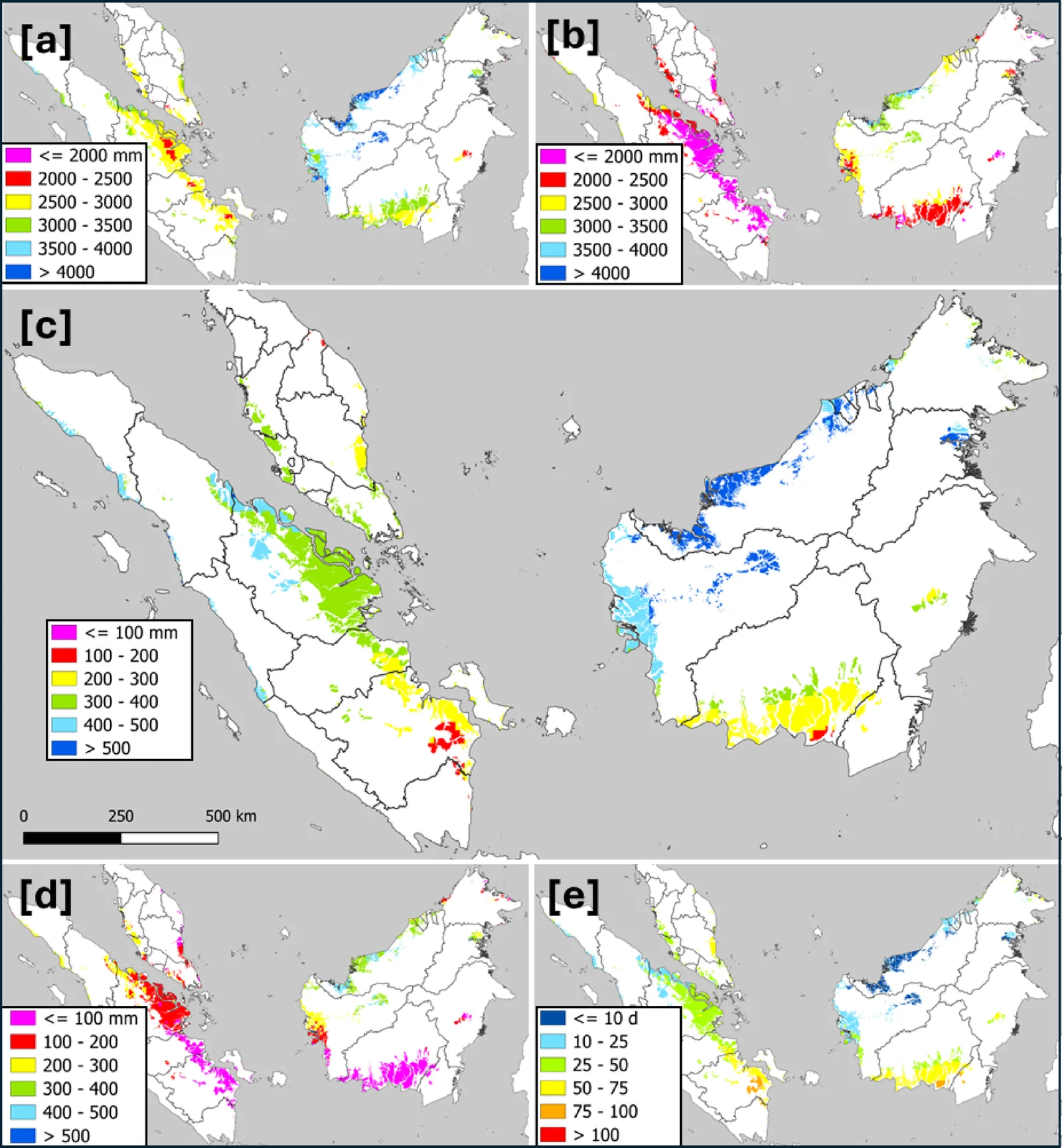
Maps of rainfall regime for tropical peatland in the study area over 2000–2024: (**a**) mean annual rainfall; (**b**) lowest annual rainfall; (**c**) mean annual 3-monthly minimum rainfall; (**d**) lowest annual 3-monthly minimum rainfall; (**e**) mean annual number of days over which 30-d rainfall was below 100 mm.

Regional rainfall variation is shown in Fig. 2; Table 2. Over the 25-year study period (2000–2024), mean annual rainfall over peatlands varied from below 3000 mm in most of Sumatra to around 4000 mm in northern Borneo i.e. Sarawak, Brunei and West Kalimantan. In the driest years on record, these numbers are generally below 2000 mm and around 3000 mm for these same regions, respectively (Fig. 2; Table 2). The mean rainfall in the driest 3-month period (which varies by area) ranges from below 40 mm along southern coastlines to above 250 mm in northern areas (Table 2). The mean annual dry period length, which for the region may be defined as the number of days with a 30-day moving total of precipitation less than 100 mm^28^, ranges from over 60 days in driest regions to less than 20 in the north (Fig. 2; Table 2).

**Table 2:**
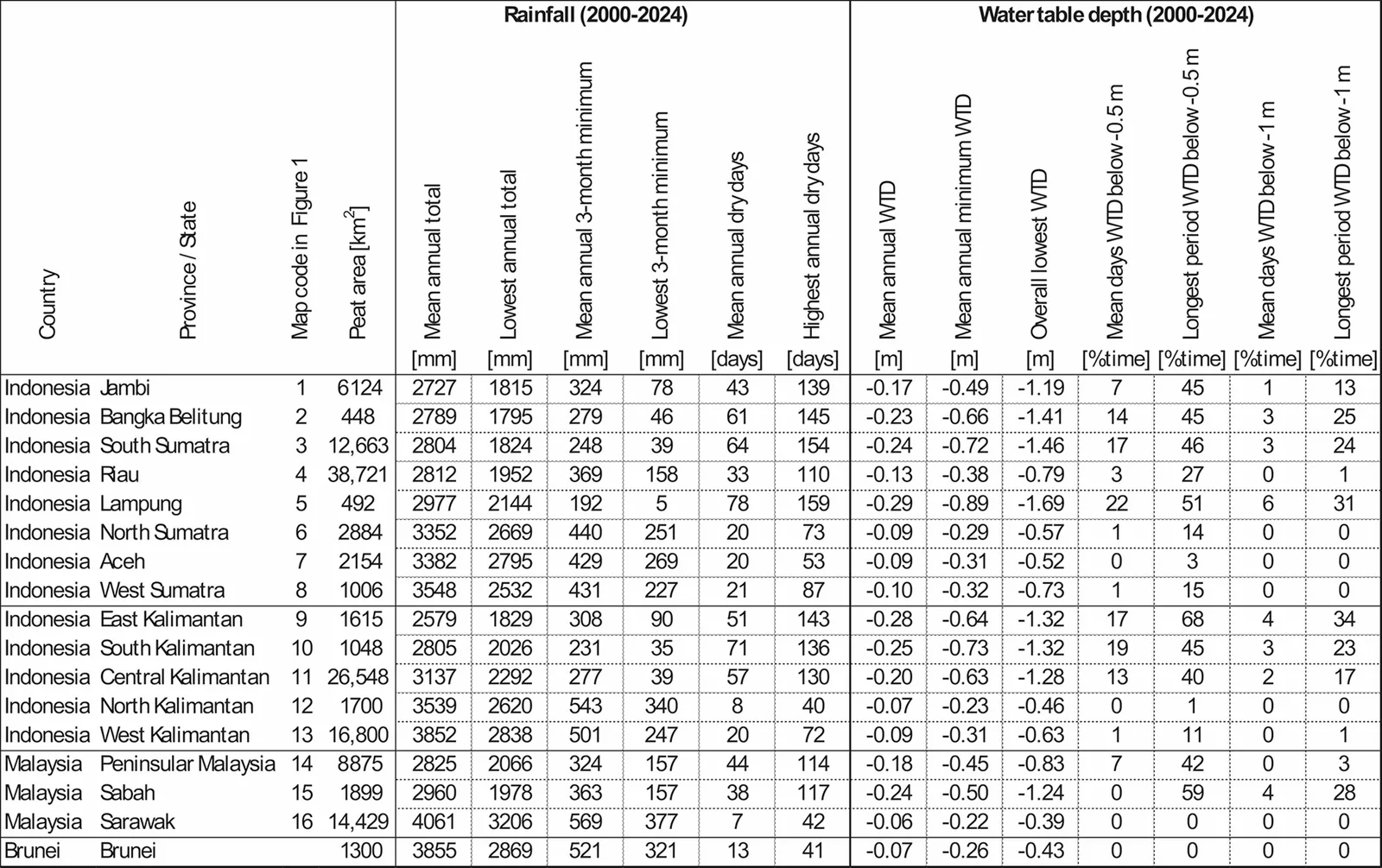
Summary statistics of regional observed annual rainfall and modelled WTD over 25-year record (2000–2024).

### Calibrated tropical forested peatland WTD model

Applying a constant *ET + WF* rate of 5 mm d^− 1^ when the water table is below the peat surface, an overall mean difference of zero between measured and modelled WTD across the 4 calibration sites was achieved applying an *ET + WF* rate of 50 mm d^− 1^ when the water table is above the peat surface, in combination with a constant *Sy* of 0.48 at all water table depths. A similar outcome could be achieved with somewhat higher or lower above-surface *ET + WF* combined with a lower or higher *Sy*, but the optimum ranges are limited to a few percent difference in parameter values.

### Differences between observed and modelled WTD at calibration sites

**Fig. 3:**
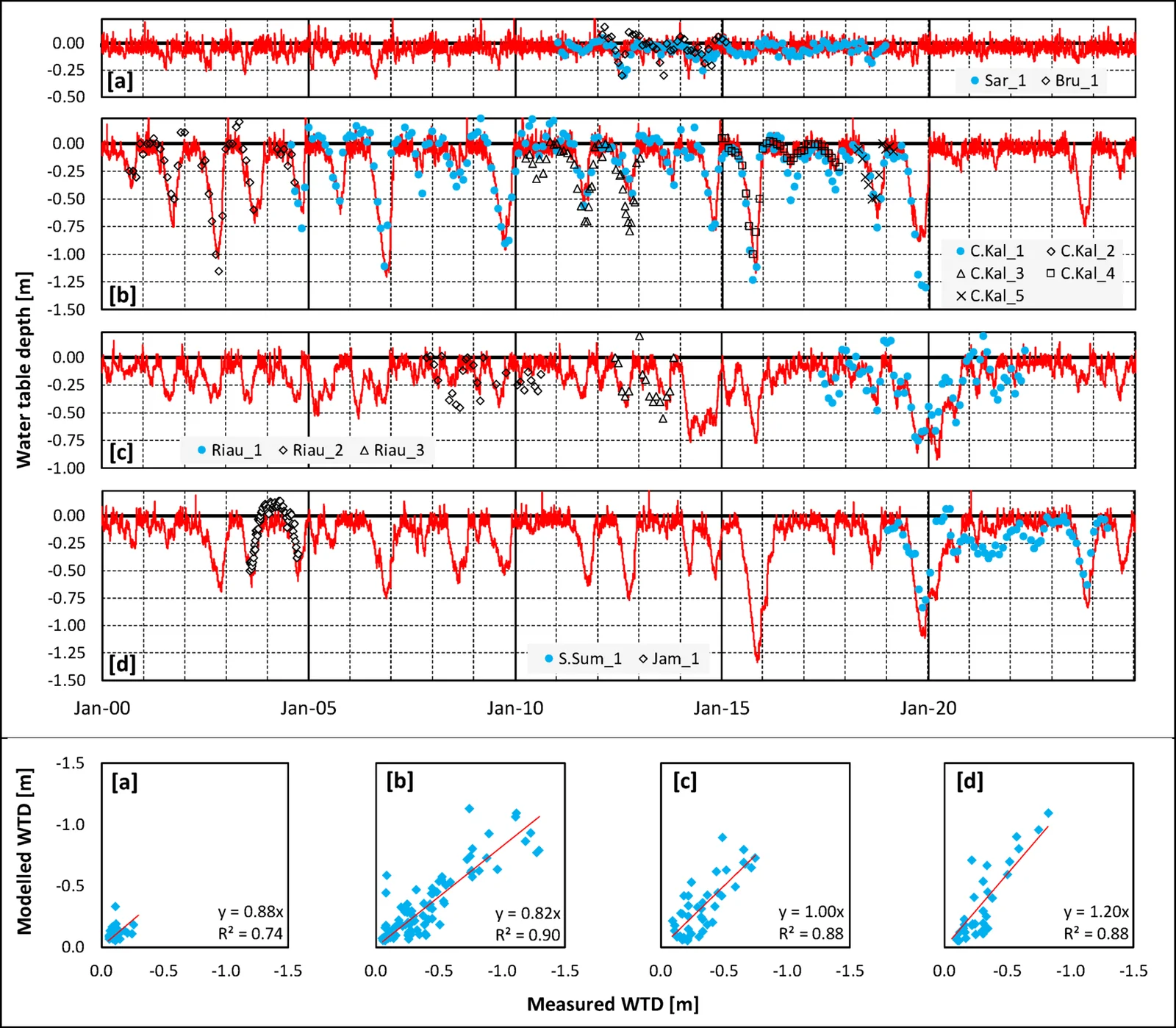
WTD model calibration results for four forested peatland sites: (**a**) Sarawak; (**b**) Central Kalimantan; (**c**) Riau and (**d**) South Sumatra. The top panel shows time series of modelled (red line) and measured (symbols) data. Blue dots present long records of field measurements that were used for calibration, as presented in Table 1. Black symbols present shorter published field records from nearby locations, that are shown for visual comparison only: Bru_1^29^, Kal_2^30^, Kal_3^31^, Kal_4^32^, Kal_5^33^, Riau_2^5^, Riau_3^34^, Jam_1^35^. The bottom panel shows regression results for the same calibration datasets. Note that the model is run on a daily basis, but calibration data are available on a monthly basis, somewhat reducing the model fit.

The R^2^ values for the relation between daily measured and modelled WTD ranged from 0.74 to 0.90 for the four calibration sites if intercepts were set at ‘zero’ (Fig. 3). Lower R^2^ values were achieved when the intercept was not set to ‘zero’, from 0.63 to 0.77 for 3 sites with the fourth site being in Sarawak where a low R^2^ of 0.04 resulted from the very limited range of WTD caused by continuously high rainfall and therefore high water levels throughout the record. Visually, the calibrated models matches most field measurements well, especially during dry periods with falling water tables (Fig. 3).

The mean of daily observed WTD values over the calibration period was in a narrow range from − 0.20 to − 0.25 m for 3 of the four calibration sites, but the fourth site in Sarawak yielded a very different value of − 0﻿.06 m (Table 1), corresponding with the much higher rainfall in that region. Modelled daily mean values differed less than 0.03 m from field measurements at any site, confirming the accuracy of the model. Differences between observed and modelled daily minimum values across the record were greatest at the South Sumatra site at 0.23 m, between − 0.82 and − 1.05 m respectively, but limited to less than 0.11 m at the other sites.

### Geographic and temporal variation in modelled WTD of forested peatlands

**Fig. 4:**
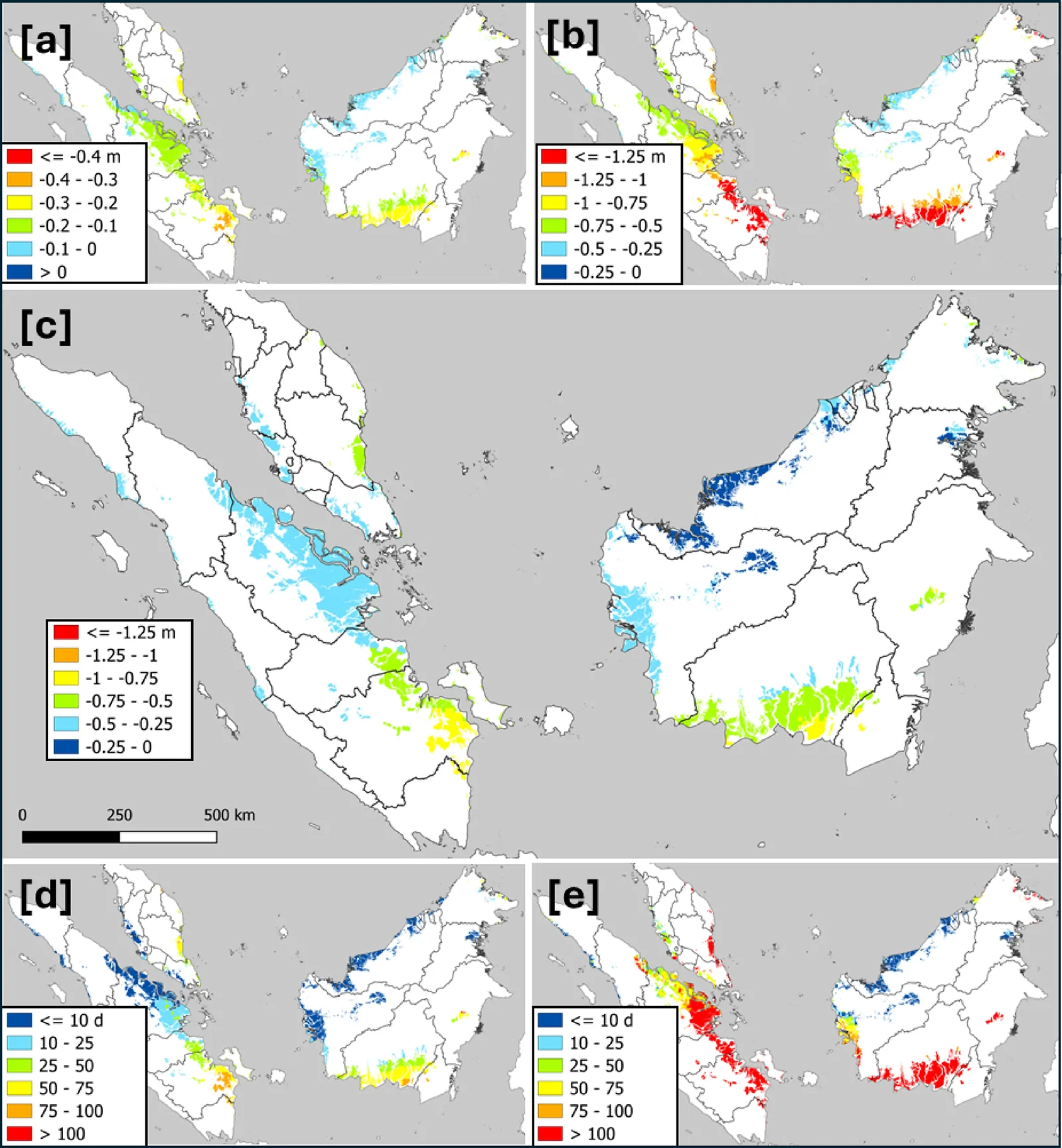
Maps of modelled ‘natural forest’ WTD in tropical peatland in the study area, 2000–2024: (**a**) mean annual WTD; (**b**) minimum WTD over 2000–2024; (**c**) mean annual minimum WTD; (**d**) mean period of time per year with WTD below − 0.5 m; (**e**) longest period of time per year on record with WTD below − 0.5 m. Note that modelled WTD also covers deforested peatlands, as this can help provide indicative WTD targets for PSF restoration.

The major spatial and temporal variation in rainfall across the region causes corresponding variation in forested peatland WTD. Mean modelled WTD is above − 0.10 m in wettest regions to around − 0.25 m in drier parts (Table 2; Fig. 4). Mean annual minimum WTD values vary accordingly, generally north to south, from ~− 0.3 to ~− 0﻿.9 m, with lowest values across the record being around − 1 m in Central Kalimantan and South Sumatra. In wettest regions the WTD is never below − 0.5 m, whereas this occurs around 15% of the time in dry regions on average and above 40% in driest years.

**Fig. 5:**
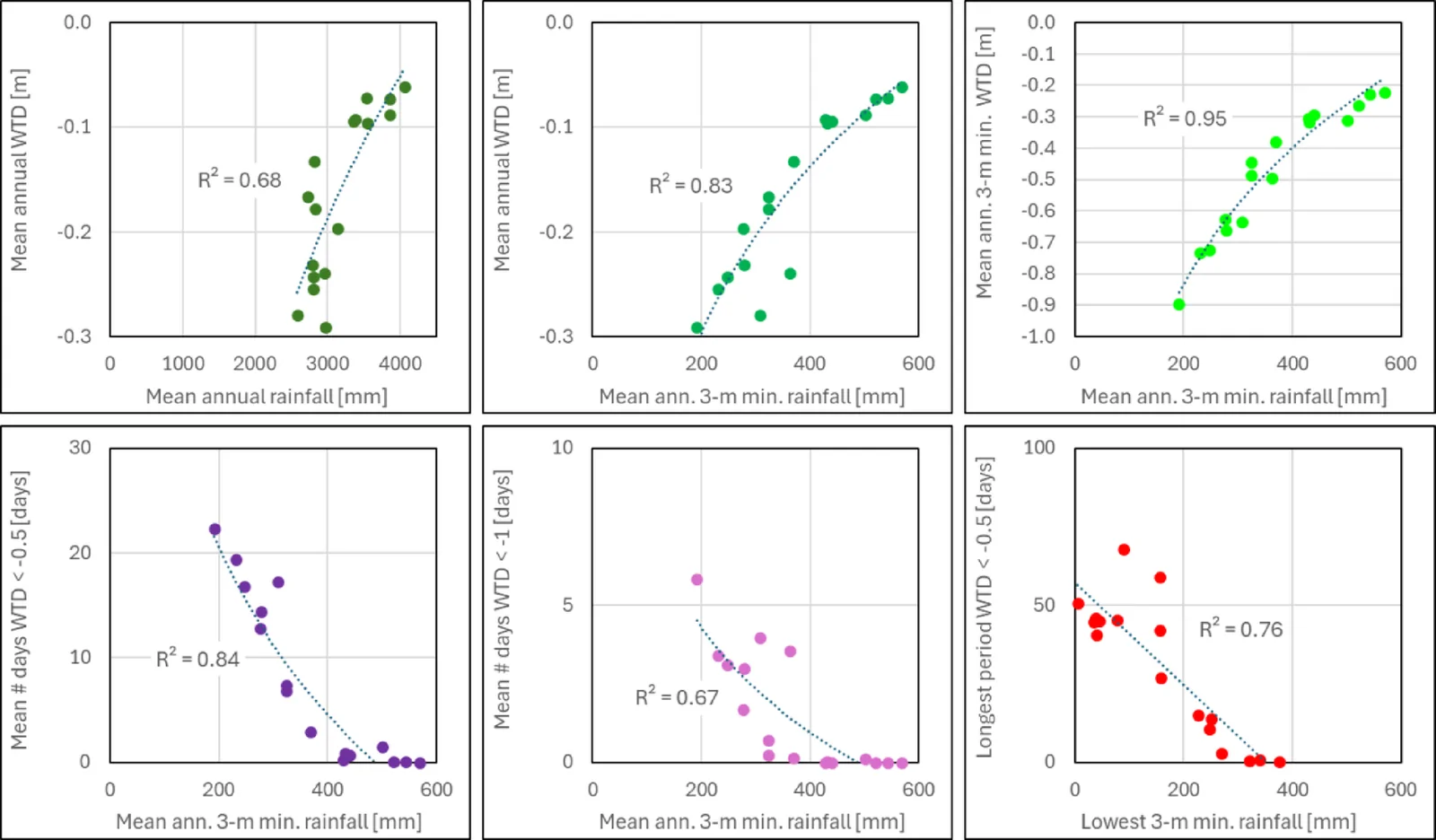
Relations between observed rainfall and modelled WTD statistics, for geographic areas shown in Table 2. Each point in the graphs presents a region in Table 2.

Regional patterns in peat ‘wetness’ are clear from most rainfall and modelled WTD parameters. However, it is worth noting that the most common descriptor of ‘wetness’, mean annual rainfall, is not very well correlated with mean annual or minimum WTD (Fig. 5). Much higher correlations are found when considering mean annual 3-month minimum rainfall, yielding an R^2^ value of 0.95 with mean annual minimum WTD.

**Fig. 6:**
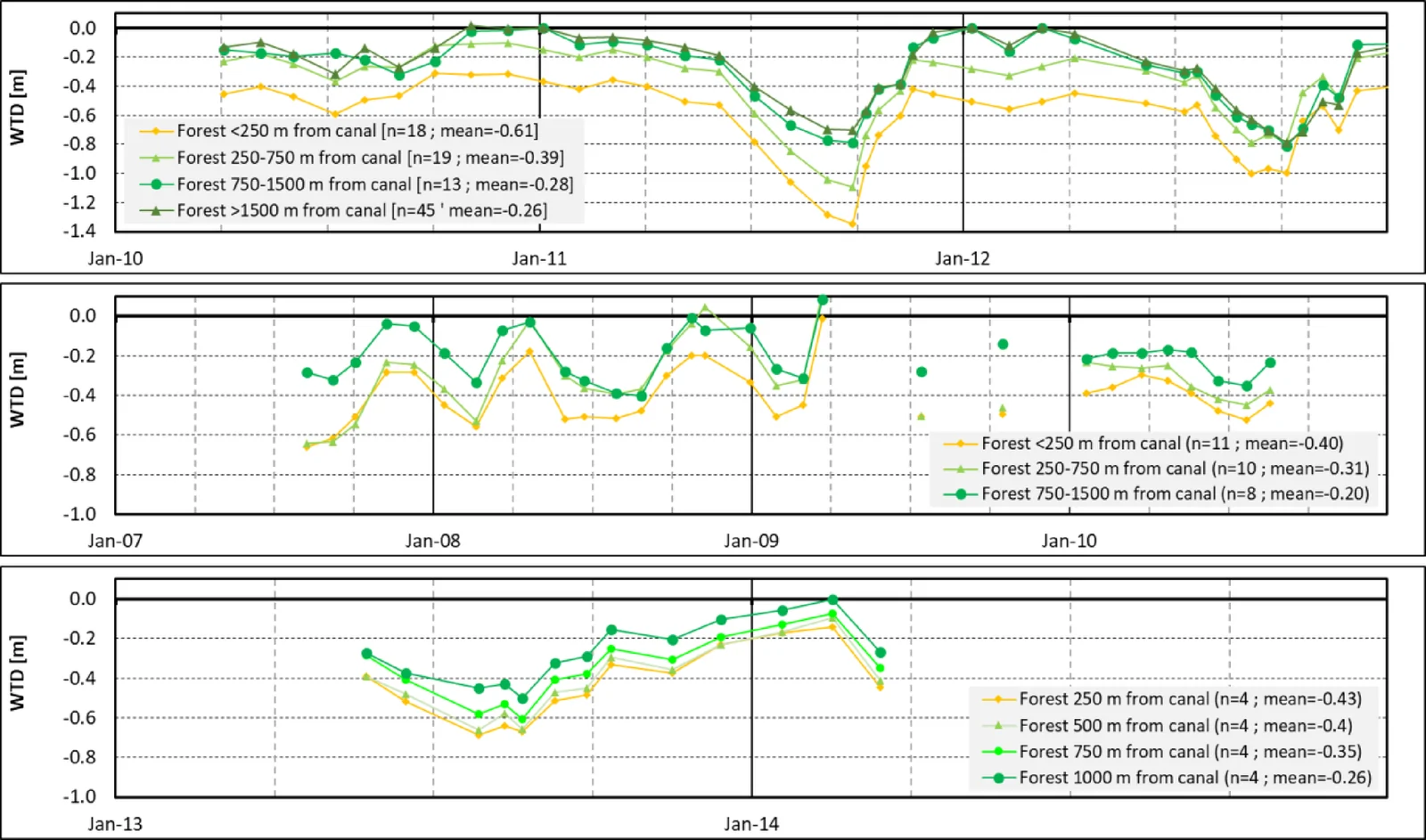
WTD records as measured at different distances from drainage features. The data presented in (**a**) and (**b**) were used and summarized in studies for sites in Kalimantan^31^ and Sumatra^5^(Indonesia) respectively; these measurements were collected along transects perpendicular to large canals, consisting of dozens of dipwells that extended up to 2200 m away from major drainage. The WTD record presented in (**c**) is in Brunei, Badas dome (Vernimmen et al., unpublished data).

## Discussion

### Representativeness of calibration WTD records for undrained conditions

The four published records used for WTD model calibration were selected on the basis of being situated in natural PSF, at least 1.5 km away from known drainage canals. Given the abundance of drainage canals, as well as ditches for logging transport, it may be that fully undrained conditions can no longer be found in Sumatra and Borneo peatlands. However, we have investigated at what distance from canals, tropical PSF may be considered ‘nearly undrained’, which is a question of wider relevance to peatland conservation and restoration projects.

The records presented in Fig. 6 indicate that water tables are consistently lowest within 250 m from canals, and still substantially below natural levels up to 750 m from canals. The difference between WTD measurements averaged over 250–750 and 750–1500 m away from canals is often less pronounced but it can still exceed 10 cm in some periods and locations. Water levels at more than 1500 m from canals differs by only a few cm from measurements over the 750–1500﻿ m range, confirming findings by Hooijer et al.^3^ who observe that a rise in canal water level after blocking by dam construction, affects WTD in PSF over 10 m in depth more than 1400﻿ m from the canal, but by less than 5 cm.

It should also be noted that field measurements of PSF WTD are generally not possible where frequent flooding prohibits access, likely resulting in an underreporting of the highest water levels in the least drained peatlands.

Considering these challenges in defining ‘natural undrained PSF WTD’ we conclude that the records used in this study are the best possible approximation of undrained natural conditions that can currently be studied in the peatlands of Sumatra and Borneo. This is also confirmed by the visual similarity of these 4 high-quality records with another 8 forest WTD records that are shown in Fig. 3, even though these additional records are not in exactly the same rainfall zone, and may be somewhat affected by drainage.

### Possible inaccuracies in WTD model rainfall input and calibration records

The accuracy of the WTD model is evaluated from its ability to approach published field measurements based on satellite rainfall input, i.e. the ‘model fit’. However, the accuracy of field WTD data is itself often limited by measurement errors, and by difficulty in defining the exact ‘peat surface level’ in uneven terrain with pronounced microtopography covered with a litter layer. Using satellite rainfall data also has inherent uncertainties; as tropical rainstorms can be very local, often covering a few km^2^ only, some individual events picked up by the satellite signals over ~ 10*10 km may not occur at the WTD measurement location, and the opposite case also occurs. It should be noted however that uncertainties would be greater using rainfall data collected on the ground, that are affected by limited gauge coverage and measurement errors as demonstrated by Vernimmen et al.^13^, apart from the generally shorter duration of such records.

Despite these limitations, we consider it unlikely that a much better model fit would be achieved if more calibration WTD records or ground rainfall data were available.

### Considerations on applying constant ET in the WTD model

Different PSF types exist across the region and even within peat domes, that have different ET response to rainfall and drought. For instance, multi-storey tall canopy forest (sometimes > 50 m) exists on lower peat dome slopes near rivers and in some wetter regions (especially North Borneo), which is very different from the pole and ‘padang’ forest with much lower canopy height (often < 15 m) that is found on top of some large peat domes. In undisturbed conditions, up to 6 distinct peat swamp forest types may be distinguished on single domes^36,37^. These forest types have different leaf area and therefore also likely different rainfall interception rates. Their root systems extend to different depths, affecting their capacity to obtain water when water tables are low, and therefore drought response. No published information on these ecological variations and hydrological interactions exists for most forest types. Moreover, rainfall seasonality is variable across the region and can be irregular and unpredictable; Riau for instance can have up to 2 moderate dry seasons per year while Central Kalimantan in most years has a single severe one and North Borneo usually has none.

For specific parts of the region, with regular seasonality and with well-studied peat and PSF like in Central Kalimantan^23^, it may be possible to create more complex models. However, in view of the variations and data limitations across the regional scale, we have chosen to assume constant ET across seasons for our model, without considering rainfall interception or water availability to roots as separate parameters. Tests with variable ET on a seasonal or event basis did not yield better results. And in practice, such variations in different aspects of hydrology and plant interaction can partly compensate each other, as is indicated for instance by the limited range in ET reported by Hirano et al.^23^ with rates for wet and dry seasons of 4.09 mm d^− 1^ and 4.60 mm d^− 1^, respectively, for Central Kalimantan. Our optimization results confirm that using a constant ET yields a sufficiently good fit for the four calibration sites to assume model validity across the study region. And the large variations that we demonstrate in regional WTD regime are clearly caused by rainfall regime as the main controlling parameter, rendering variations in ET of secondary importance.

### Representativeness of calibration sites

While the four calibration records were selected based on strict criteria of duration, peat thickness, distance to canals and overall thoroughness of research set-up, that in itself may not guarantee representativeness for regional conditions. However, Fig. 3 shows that WTD records collected at nearby sites indicate very similar fluctuations and mostly match the calibrated model well even though these sites are tens of kilometres away from calibration sites, a few hundred kilometres for the Brunei site. This confirms that regional geographic variation in rainfall is indeed the main control on hydrological regime, and demonstrates that calibration records are representative for the regions they were collected in.

It is worth noting that while the calibration sites were on peat deeper than 5 m, the limited role of groundwater flow in the peat water budget suggests that the model will be applicable to shallow peat as well as long as the forest vegetation, fibric top peat and hummocky peat surface microtopography that control tropical peatland hydrology are intact. Moreover, we observe that remaining forest in especially Sumatra, and therefore forest conservation and restoration potential, is concentrated on deep peat^38^.

### Changes in rainfall and WTD over time

From Fig. 3 it can be seen that at two of the four calibration sites, the three occurrences of lowest water tables and also the longest periods with WTD below − 0.5 m occurred since 2014, i.e. in the last half of the 25-year modelling period, with the El Niño related 2015 and 2019 drought events being the most severe. While this pattern is not as clear for the other two calibration sites, it appears that in Sumatra at least, drought conditions that affect peatland functioning may have become more common in recent years. On the other hand, the years of 2020, 2021, 2022 and 2024 have some of the highest mean water tables on modelled record in the Central Kalimantan site and to a lesser extent the Sumatra sites, possibly indicating a trend towards greater year-to year and seasonal variation rather than towards just drier conditions. While a full assessment of potential changes in rainfall patterns across SE Asia is beyond the scope of this study, and no such research is published specifically for Sumatra and Borneo, we propose that peatland restoration projects should be planned in the expectation that prolonged droughts may become more common in future, as indicated for Southeast Asia as a whole by several studies^39,40^. This would require more redundancy in peatland rewetting measures than may seem necessary under current conditions.

### Implications for PSF conservation and restoration

The maps of modelled WTD resulting from this study may be used to provide an optimum target WTD for conserving remaining PSF. Where the actual WTD as measured in a PSF area, over a period of several years, is found to be substantially below the modelled WTD for that area in terms of mean or minimum values, measures may be required to reduce drainage and raise water levels. For instance, in large areas in Central Kalimantan water levels in canals were found to be more than 1 m below the peat surface many years after canal blocking in restoration projects^41^, suggesting blocking had failed to sufficiently raise water levels.

For deforested or burnt drained peatlands where restoration to PSF is planned and involves rewetting, it is usually best to not raise water levels too much too soon, as inundation should be prevented at the initial tree growth stage. A recent study by Hooijer et al.^3^ found that water levels in parts of a restoration area were raised to the point of frequent flooding, preventing nearly any tree growth. The initial WTD target applied in rewetting design may therefore be somewhat below the optimum WTD as represented by model results for undrained PSF. Continued peat surface subsidence^5^ after the initial rewetting will ensure that the optimum WTD will be reached gradually over the years as hydrology and vegetation establish a new balance. It has been proposed that a pragmatic generic short-term target for restoring water levels in drained peatlands could be a mean (not minimum) WTD of − 0.3 to − 0﻿.4 m^3^. Such caution is also warranted in view of findings by Kreyling et al.^42^ indicating that rewetting of temperate peatlands often produces hydrological and soil results that differ from original conditions, suggesting that the natural WTD values presented by us could be considered an indicative aim of rewetting tropical peat swamps rather than a precise target.

It is furthermore worth noting that except for a relatively limited peatland area in Northern Borneo, water tables in forested undrained peatlands in all regions fall well below − 0.5 m, frequently and for extended periods. This implies that restoration targets aiming to maintain water tables above − 0.4 m at all or most times would not be met even in most natural undrained PSF, and appear even less realistic for drained peatlands.

## Conclusions

The spatial and temporal variation in rainfall and WTD regimes in peatlands along the coastlines of Sumatra and Borneo is found to be such, that studies into carbon cycle, fire risk, ecosystem conditions or management targets should be interpreted considering that not all sites have equal WTD in natural conditions. Notably, studies in northern Borneo relate to conditions of near-constant rainfall and high water tables that are very different from most peatlands in SE Asia, but substantial variations exist within the Island of Sumatra as well.

The study presented here is meant to provide reference input to research and management of peatlands in SE Asia. Assessments of the effectiveness of water management interventions in peatland rehabilitation areas or plantations, in reducing carbon emissions or surface subsidence, will benefit from understanding what the WTD would have been in a ‘natural’ state. It will be better to use the data presented here as a benchmark than to simply assume that water tables in natural peatlands are always near the surface, which we show not to be the case in most of the region.

## Data Availability

All data used are available in the public domain, with sources indicated in this article. The rainfall and WTD maps presented in this paper are available online in GIS format (https://doi.org/10.17632/69mbg22fxf). To ensure that the published WTD maps described here cover the period of field measurements in ongoing studies, and allow comparison in upcoming publications, updated maps can be shared by the authors upon reasonable request.
